# Value of Scientific Services

**Published:** 1859-10

**Authors:** 


*Value of Scientific Services.*—It is a most common complaint among scientific men, that when called upon to do any service for the public, their labors are generally miserably paid, it being thought sufficient to have the honor of doing the work. It has become necessary for them either to take the ground that they will not perform these services, which many do, or for some one to take the initiative in making the value of scientific labor appreciated. We are glad to see that the example. has now been set by Prof. Doremus, of this city. For his chemical analysis in the Stephens case, in which two entire bodies were analyzed, and which was by far the most complete investigation ever attempted, he has charged and received $3,000 for his services, and $800 for new apparatus. This analysis will be of incalculable value to justice, and will be referred to as long as law and science exist. We are gratified to see that such a service has been appreciated ; though for the amount of actual labor expended, which we have not space to detail, the pay is certainly not too much, if, indeed, it be sufficient. We hope at some future time to be able to present to our readers an account of the medical points in this interesting case.

